# lncRNA Ubr5 promotes BMSCs apoptosis and inhibits their proliferation and osteogenic differentiation in weightless bone loss

**DOI:** 10.3389/fcell.2025.1543929

**Published:** 2025-04-02

**Authors:** Dong Wang, Yuan Gao, Yingjun Tan, Na Li, Xi Li, Jiaxiang Li, Yikai Pan, Xingcheng Zhao, Ming Yan, Yongchun Wang

**Affiliations:** ^1^ Department of Aerospace Medical Training, School of Aerospace Medicine, Air Force Medical University, Xi’an, China; ^2^ State Key Laboratory of Space Medicine Fundamentals and Application, China Astronaut Research and Training Center, Beijing, China; ^3^ Department of Orthopedic Surgery, Xijing Hospital, Air Force Medical University, Xi’an, China

**Keywords:** weightless bone loss, lncRNA-mRNA coexpression network, simulated weightlessness, bone marrow mesenchymal stem cells, lncRNA Ubr5

## Abstract

**Background:**

Weightless bone loss is a common pathological phenomenon in weightless environments, yet its specific molecular mechanism remain incompletely elucidated. The aim of this study was to systematically investigate the differential expression profiles of mRNAs and long noncoding RNAs (lncRNAs) to explore the molecular pathogenesis underlying weightless bone loss.

**Methods:**

Transcriptome sequencing was performed on bone marrow mesenchymal stem cell (BMSCs) samples from the Ground control group and simulated microgravity (SMG) group using Illumina technology. Using the DESeq2 algorithm, we accurately identify and analyzed the differentially expressed genes (DEGs). Subsequently, the molecular functions and signaling pathways enriched by DEG were comprehensively analyzed by GO and KEGG. In addition, by constructing lncRNA-mRNA coexpression network, this study screened and verified key lncRNAs as potential genes to further explore their role in the occurrence and development of weightless bone loss.

**Results:**

A total of 215 differentially expressed lncRNAs (DElncRNAs) and 381 differentially expressed mRNAs (DEmRNAs) were identified, in the SMG group. DEmRNAs were primarily involved in the cell response to mechanical stimulation, microtubule motility and TNF signaling pathway. Meanwhile, DElncRNAs are significantly enriched in cell differentiation, fatty acid metabolic process and biosynthesis of amino acids. In addition, the expression levels of related lncRNAs and mRNAs in weightless bone loss were verified via qRT-PCR. lncRNA-mRNA coexpression network found that lncRNA Ubr5 closely related to osteoblast proliferation and differentiation. Further experimental results revealed that knocking down lncRNA Ubr5 can promote the apoptosis of BMSCs and inhibit their proliferation and osteogenic differentiation.

**Conclusion:**

This study revealed the molecular pathogenesis of weightless bone loss, identified lncRNA Ubr5 as a potential intervention target, and provided an important scientific basis and strategic guidance for the prevention and treatment of weightless bone loss.

## Introduction

Weightless bone loss is a specific type of disuse osteoporosis (Takata and Yasui, 2001), characterized by a systemic degenerative bone disease that results from the loss of gravity. The condition marked by a loss of bone mass and a decrease in bone density, accompanied by the destruction of the bone tissue microstructure and an increase in bone fragility, ultimately triggers fragility fractures. Spaceflight data show (Vico et al., 2000; Smith et al., 2005) that weightlessness-affected bone density decreases by an average of 1.0%–2.0% per month, and this bone loss is site-specific and selective and is mainly concentrated in weight-bearing bones, such as the femur and spine. Importantly, weightless bone loss lacks self-limitation and gradually intensifies with increasing weightless exposure time, which severely affects the smooth implementation of space missions (Iolascon et al., 2013). To prevent the risk of osteoporosis and fragility fractures associated with long-term space residence, various interventions have been explored. These include nutritional supplements, physical exercise and drug interventions. However, the effectiveness of these measures has been limited, and the problem of bone loss still exists, which threatens the health of astronauts (Qin et al., 2020; Karsenty and Mera, 2018; White and Averner, 2001). Therefore, investigating the effects of weightlessness on the human skeletal system and its underlying molecular mechanisms has become an important topic in aerospace medical research.

Long noncoding RNAs (lncRNAs) represent a class of nonprotein-coding RNA molecules that are more than 200 nucleotides in length. Although lncRNAs do not encode proteins, they play important regulatory roles in cells in processes such as cell differentiation, gene expression, and the occurrence and development of various diseases (Kung et al., 2013; Statello et al., 2021). Increasing evidence has shown (Wang and Kong, 2018; Wicik et al., 2021) that lncRNAs play important roles in the pathogenesis of osteoporosis, affecting bone metabolic processes through multiple mechanisms, including the regulation of bone marrow mesenchymal stem cells (BMSCs) (Hu et al., 2023), osteoblasts (Hou et al., 2023) and osteoclasts. For example, (Zhao et al., 2024), studies have shown that the lncRNA GM15416 regulates the apoptosis and differentiation of osteoblasts through the c-Fos/Fas axis. Reduced expression of GM15416 decreases c-Fos/Fas levels, thereby inhibiting the apoptosis of osteoblasts and promoting osteogenesis. In addition (Han et al., 2021), lncRNA H19 enhances the osteogenic differentiation of BMSCs by activating the Wnt/β-catenin signaling pathway, suggesting its potential as a preventive factor and potential therapeutic target for osteoporosis. More importantly (Liu et al., 2022), the mechanosensitive lncRNA Neat1 was found to regulate osteoblast function through a paracrine blockade-dependent Smurf1 mRNA, which is particularly critical in response to simulated weightlessness signals. However, the current research on the correlation between lncRNAs and weightless bone loss remains limited. Further exploration is needed to identify more therapeutic targets that could effectively modulate weightless bone loss and provide novel strategies for mitigating this condition.

In this study, we analyzed the differentially expressed lncRNAs (DElncRNAs) and mRNAs (DEmRNAs) in the ground and simulated microgravity (SMG) groups *via* high-throughput sequencing. The lncRNA-mRNA coexpression network (Langfelder and Horvath, 2008) was subsequently constructed, and the functional roles of the DElncRNAs were predicted via analysis of the coexpressed DEmRNAs. Gene Ontology (GO) and Kyoto Encyclopedia of Genes and Genomes (KEGG) enrichment analyses (Wang et al., 2022) were performed to further elucidate the biological functions of the differentially expressed genes. To verify our sequencing results, we measured the expression levels of the DEmRNAs and DElncRNAs via qRT-PCR. We subsequently constructed a lncRNA-mRNA coexpression network related to osteoblast proliferation and differentiation and selected ubiquitin protein ligase E3 component n-recognin 5 (Ubr5) as the target gene for follow-up studies. Finally, we conducted *in vitro* experimental studies on the biological function of Ubr5. This study provides valuable insights into the molecular mechanism of weightless bone loss and provides a reliable reference for identifying potential therapeutic targets for weightless bone loss.

## Materials and methods

### Tissue samples from disuse osteoporosis patients

Owing to the limited number of astronauts and the ethical issues of exploratory experiments, *in vivo* weightlessness experiments have been limited. Therefore, we selected patients who were chronically bedridden with lumbar spine disease and who required lumbar spine surgery from August 2020 to November 2022 in the Department of Orthopedics of the First Affiliated Hospital of the Air Force Military Medical University as the study subjects. According to the inclusion and exclusion criteria, 6 patients with disuse osteoporosis and 6 patients with normal bone mineral density were included. Fresh lumbar spinous specimens were removed intraoperatively and stored at −80°C (Supplementary Figure S1). The expression of the lncRNA Ubr5 in bone tissue was subsequently determined via qRT-PCR. This study was approved by the Medical Ethics Committee of the First Affiliated Hospital of Air Force Military Medical University (approval document KY20233212-1). The basic information of the patients is shown in Supplementary Table S1.

### Establishment of an SMG mouse model

Male C57BL/6 mice provided by the Laboratory Animal Center of Air Force Military Medical University were used in this study. All animal experiments were approved by the Experimental Animal Ethics Committee of the Air Force Medical University. Twelve 6-week-old male mice (160 ± 20 g body weight) were selected and randomly divided into a tail-suspended hindlimb unloading group (TS group) and a control group (CON group). In the TS group, each mouse tail was washed and air-dried, coated with a rosin tincture, and secured with three circular tapes. The mouse was subsequently suspended in a glass box of 16 cm × 30 cm×28 cm with a traction rope tied to a stainless steel rod. The body of each mouse was angled at 30°C with the ground and could be freely rotated, with the tail being suspended for 4 weeks. In the CON group, the mice were reared in a single cage in the same environment without tail suspension. During the 4-week modeling period, both groups of mice were allowed to eat and drink freely. During the experiment, the daily activities of the mice (such as eating, drinking and behavior) were recorded to ensure their health. The SMG mouse model is shown in Supplementary Figure S2.

### Establishment of a BMSCs culture and simulation of a weightlessness model

The BMSCs were cultured in DMEM containing 10% FBS and 1% penicillin-streptomycin in a 5% CO_2_ incubator at 37°C, and a 2D-gyroscope was used to simulate the weightlessness of the cells. The day before simulated weightlessness, the cells were inoculated on 2.55 cm × 2.15 cm slides at a density of 1 × 10^5^ cells per sample and cultured overnight in a six-well plate. When the cell density on the slide reached approximately 50%, the inoculated cell slide was inserted into the stainless steel bracket of the rotary machine and placed into the preheated rotary chamber filled with DMEM, and the osteogenic differentiation fluid BMP-2 was added for osteogenic induction. The slide was then placed on the rotating arm of the rotary machine after the bubbles were removed. The mixture was rotated horizontally in a cell incubator at 24 r/min to simulate weightlessness for 48 h. The cells in the ground group were placed next to the gyroscope and incubated for the same amount of time. The cells were collected in a centrifuge tube, and RNA and proteins were subsequently extracted. The 2D-gyroscope is a kind of laboratory equipment that simulates microgravity environment. The sample is rotated at high speed around an axis perpendicular to the gravitational field. When the rotation speed is fast enough, the centrifugal force generated by the rotation of the rotator can be offset by gravity, and the sample in the rotating system no longer senses the rapidly rotating gravity vector, thus achieving the purpose of simulating the weightlessness effect. The 2D-gyroscope structure is shown in Supplementary Figure S3.

### RNA extraction, library construction and sequencing

The total RNA of the ground and SMG samples was extracted *via* a Trizol kit and evaluated for quality control. Ribosomal RNA was removed, and the obtained RNA was randomly interrupted into short fragments. The first strand of cDNA was synthesized by using the fragmented RNA as a template with a six-base random primer. The second strand of cDNA was synthesized by adding buffers, dNTPs, RNase H and DNA polymerase I. The second strand of cDNA was purified via the QiaQuick PCR Kit, eluted with EB buffer, end-repaired, added with base A, and added to sequencing junctions, and then, the second strand was degraded by the UNG enzyme. Fragment size selection was performed via agarose gel electrophoresis, PCR amplification was performed, and finally, amplification and sequencing were performed via an Illumina NovaSeq 6000 sequencer.

### Sequencing data quality control and ribosome comparison

To ensure data quality, we filtered the original data before information analysis to reduce the interference of invalid data analysis. The raw reads were controlled by fastQ to remove the adapter-containing reads, the reads with N ratios >10%, and the low-quality reads with Q ≤ 20 and the number of bases exceeding 50% of the whole reads, and we generated clean reads for further analysis. Then, we used the short-read matching tool Bowtie2 to match the clean reads to the ribosomal database of the species, removed the reads that were matched to the ribosomes without allowing mismatches, and retained the unmapped reads for subsequent transcriptome analysis.

### DElncRNA and DEmRNA screening and analysis

To compare the gene expression levels between the ground and SMG groups, we used DESeq2 software to conduct differential expression analysis of mRNAs and lncRNAs between the ground and SMG groups. First, we standardized the read count. The hypothesis testing probability (p value) was subsequently calculated according to the model. Finally, multiple hypothesis testing correction was performed to obtain the false discovery rate (FDR) value. On the basis of the difference analysis results, lncRNAs and mRNAs with FDR <0.05 and |log2FoldChange (FC)| > 1 were identified as significantly different lncRNAs and mRNAs.

### Enrichment analysis of DElncRNA and DEmRNA gene functions and signaling pathways

DAVID (http://david.abcc.ncifcrf.gov/) gene functional annotation online database tools and R language were used to perform GO and KEGG analyses on differentially expressed genes (DEGs) that had been screened to explore the biological significance of DEGs at the molecular level. GO is an international standard classification system for gene function that includes mainly cellular component (CC), molecular function (MF), and biological process (BP) terms. KEGG is the main public database of signaling pathway information that can be used to analyze signaling pathways related to DEGs.

### LncRNA-mRNA interaction network construction and correlation analysis

Since lncRNAs themselves have no function, to study the potential function of DElncRNAs and the interaction between lncRNAs and mRNAs, we used the trans analysis method to correlate lncRNAs and mRNAs. The basic principle of trans target gene prediction is that the function of lncRNAs is not related to the location of the coding gene but is related to the protein-coding gene coexpressed with the lncRNA. Target genes can be predicted by correlation analysis or coexpression analysis of lncRNAs and protein-coding genes between samples. When the sample size was greater than or equal to 6, the Pearson correlation coefficient method was used to analyze the expression correlation between lncRNAs and protein-coding genes among the samples. Protein-coding genes with absolute correlation values greater than 0.999 were selected for GO/pathway functional enrichment analysis to predict the main functions of the lncRNAs. A coexpression network of lncRNA-mRNA transcripts was constructed, and the lncRNA-mRNA correlation network was mapped via Cytoscape software.

### qRT-PCR experiment

The cell samples were collected, total RNA was extracted from each sample via Trizol reagent, and the concentration and purity were measured. The first Strand cDNA Synthesis Super Mix for qPCR was used to reverse transcribe cDNA, and the cDNA mixture obtained via reverse transcription was amplified repeatedly via the qPCR SYBR Green Master Mix reaction system. With GAPDH as the internal reference gene, the expression of related genes was determined with a LightCycler 480 detection system. All sequences of primers used in this study are listed in Table 1.

**TABLE 1:** Primer sequences.

Primer name	Primer sequence (5′–3′)
M-OCN-F	CCTGAGTCTGACAAAGCCTTCAT
M-OCN-R	GGCAGCACAGGTCCTAAATAGTG
M-OPN-F	TTTCACTCCAATCGTCCCTACAG
M-OPN-R	TCCTTAGACTCACCGCTCTTCAT
M-BAX-F	CGTGGTTGCCCTCTTCTACTTTG
M-BAX-R	CCTTCCTAATGCCAACCTGTGAA
M-Bcl-2-F	GGATTGTGGCCTTCTTTGAGTTC
M-Bcl-2-R	CTTCAGAGACAGCCAGGAGAAAT
M-PCNA-F	TCCTTAGACTCACCGCTCTTCAT
M-PCNA-R	AGGTACCTCAGAGCAAACGTTAG
M-Creb5-F	GCAGCGTTGTGATTCAGCAAGC
M-Creb5-R	TCTGGCGGTTGGTGGAAGGAG
M-Ackr3-F	TCAGCATCAGGCGACCAGGAG
M-Ackr3-R	GCAGCCAACATACCAGGAAGACC
M-Fsip1-F	CAGTGCGGTTGGCTGGTTCC
M-Fsip1-R	AGGCTGAAGATGGTGGGAGAGTC
M-Pcsk9-F	AGCAGCCAGGTGGAGGTGTATC
M-Pcsk9-R	GCCTGTCTGTGGAAGCGTGTC
M-Aspn-F	GTGTGCTCTGCCAAACCCTTC
M-Aspn-R	GCTCCCAAATTCCGCTTACCC
M-Atoh8-F	CGTGGAAGACTGTGTGCGTTAAAG
M-Atoh8-R	GGCGGTGGTGCTGACTGTG
M-Itih4-F	TCTGGCGGGCAACTGAAGG
M-Itih4-R	TGAAGGCAAGAACAGCACAAGG
M-Dnhd1-F	GTGTGCTCTTGTCTGGTGTAGTG
M-Dnhd1-R	TTGTCTCTTGCTGTAGGTGGTATTC
M-lncRNA Tshz1-F	TATTCTCAGAGTCCTGCTGGTCTA
M-lncRNA Tshz1-R	CTGGCTGGCATCGTTGTTG
M-lncRNA Lmo7-F	CCTCCAGGTAGCCACAGAAG
M-lncRNA Lmo7-R	GATGTCACTTTCGCAACCTTCC
M-lncRNA Srsf10-F	CAGGCTTGAAGAGAAGGGAGAG
M-lncRNA Srsf10-R	TGTGGATAAATCGAGGCTTTGTCT
M-lncRNA Ttll3-F	TACGCCGAGGCTGCTATTC
M-lncRNA Ttll3-R	TACGCCGAGGCTGCTATTC
M-lncRNA Epc1-F	CCCAATAAACGCACGGCATAT
M-lncRNA Epc1-R	CTGAATCTACAGAGGATGAGGAGG
M-lncRNA Ints8-F	TGTTCGGGAGGACATTGTGAAT
M-lncRNA Ints8-R	CGGATTGGAGGTTAGGTATGGAA
M-lncRNA Ifnar1-F	CGTGTCAGAGCAGAGGAAGG
M-lncRNA Ifnar1-R	CGGGAGGAGAGATGTGGACTA
M-lncRNA Ubr5-F	CAGCACCTCCACCATTCCA
M-lncRNA Ubr5-R	AGAACCGCACTGTCGCATA
H-lncRNA Ubr5-F	TCAAGCGTTGGTTCTGGTCA
H-lncRNA Ubr5-R	AGGGCATAGGCTGGAATCCT
M-GAPDH-F	GGTGAAGGTCGGTGTGAACG
M-GAPDH-R	CTCGCTCCTGGAAGATGGTG
H-GAPDH-F	CAGGAGGCATTGCTGATGAT
H-GAPDH-R	GAAGGCTGGGGCTCATTT

### Cell transfection

The cells were inoculated in six-well plates and transfected when the cell confluence rate reached 40%–50%. We targeted the lncRNA Ubr5 with negative control oligonucleotides and short interfering RNA (siRNAs). Lipofectamine 2000 reagent (Invitrogen, United States) was used to introduce NC and siRNA into the cells in strict accordance with the manufacturer’s instructions. The best lncRNA Ubr5 siRNA was identified for subsequent experiments. The siRNA sequences used were as Table 2.

**TABLE 2:** Primer sequences (siRNA).

Primer name	Primer sequence
lncRNA Ubr5-1-as	UCAUCAUUUCUCGUAAUCUTT
lncRNA Ubr5-1-ss	AGAUUACGAGAAAUGAUGATT
lncRNA Ubr5-2-as	AAACUGGAUCCUGGUGGUCTT
lncRNA Ubr5-2-ss	GACCACCAGGAUCCAGUUUTT
lncRNA Ubr5-3-as	GGUCAUUCCUCUUGCAUCCTT
lncRNA Ubr5-3-ss	GGAUGCAAGAGGAAUGACCTT
lncRNA Ubr5-4-as	UUUCUAGGAUGGUGAUUGCTT
lncRNA Ubr5-4-ss	GCAAUCACCAUCCUAGAAATT

### Western blot analysis

The samples from the ground and SMG groups were collected, and total protein was extracted by adding appropriate amounts of cell lysate and phenylmethylsulfonyl fluoride (PMSF), respectively. A Pierce BCA assay kit (Beyotime Biotechnology, China) was used to determine the protein standard curves and protein concentrations of the two samples. The protein samples were then mixed with buffer solution and boiled at 100°C for 30 min. The proteins were separated via 10% sodium dodecyl sulfate-polyacrylamide gel electrophoresis (SDS-PAGE) and transferred to polyvinylidene fluoride (PVDF) membranes via the semidry transfer method. The membrane was blocked with 5% skim milk, Tris-buffered saline and Tween 20 (TBST) buffer for 2 h at room temperature; subsequently, the membranes were incubated with primary antibodies at 4°C overnight. Goat anti-rabbit horseradish peroxidase conjugate (Abcam, United Kingdom) was incubated at room temperature for 1 h. Finally, the protein signal was detected with an enhanced chemiluminescence kit, and the band density was measured via the Viber Bio imaging tool.

### EDU analysis

Cell proliferation was analyzed using EDU kit. BMSCs were inoculated on slides and placed in six-well plates. After simulated weightlessness for 48 h, 1 mL EDU solution was added to each well and incubated for 2 h. Cells were then fixed with 4% paraformaldehyde for 30 min, incubated with 0.2% glycine for 10 min, and permeated with 0.5% Triton-X100 for 10 min. Subsequently, each well was added with 600 μL Apollo staining reaction solution, incubated at room temperature and away from light for 30 min, and washed with 0.5% Triton-X100 penetrant for 10 min. Finally, 1 mL 1X Hoechst reaction solution was added to each well and incubated at room temperature for 30 min away from light. The stained cells were imaged using a fluorescence microscope.

### Alkaline phosphatase (ALP) staining

The BMSCs were inoculated on slides, transfected and subjected to simulated weightlessness for 48 h. The slides with inoculated cells were removed and placed on a clean six-well plate, fixed with 4% paraformaldehyde for 15 min, and washed 3–5 times with PBS for 3–5 min each. Subsequently, 1 mL of BCIP/NBT staining working solution from the BCIP/NBT ALP kit was added per well and incubated for 24 h at room temperature in the dark. After the staining reaction was terminated, the sample was dried at room temperature and stored in the dark. The blue-purple color indicates ALP activity.

### Flow cytometry

Each group of cells was digested and centrifuged and then washed with PBS once, after which the cells were resuspended in 1 mL of binding buffer and centrifuged at 300 xg for 10 min, after which the supernatant was discarded. The cells were then resuspended in 1 mL of binding buffer such that the cell density reached 1 × 106 cells/mL. A total of 100 μL of cells was added to each EP tube, 5 μL of Annexin-V-FITC was added, the mixture was mixed well, and the mixture was incubated for 10 min at room temperature in the dark. Five microliters of PI was added, and the mixture was incubated at room temperature in the dark for 5 min. Then, 400 μL of PBS was added, and the mixture was mixed well, stored at 4°C, and tested by flow cytometry within 1 h. The negative control tubes were incubated without Annexin V-FITC and PI. After flow cytometry, the results are expressed as the percentage of apoptotic cells. In the figure, B1 represents necrotic cells, B2 represents late apoptotic cells, B3 represents normal cells, and B4 represents early apoptotic cells. The percentage of apoptotic cells was B2+B4.

### Statistical analysis

SPSS 26.0, ImageJ, and GraphPad Prism 8.0.2 were used for statistical analysis. All experimental data are presented as the means ± standard deviations. Student’s t-test was used for statistical analysis of two groups of unpaired data, and one-way ANOVA was used for multiple groups of unpaired data. All the experiments were repeated three times under the same conditions, and P < 0.05 was considered to indicate statistical significance.

## Results

### LncRNA sequencing data description and identification

**FIGURE 1:**
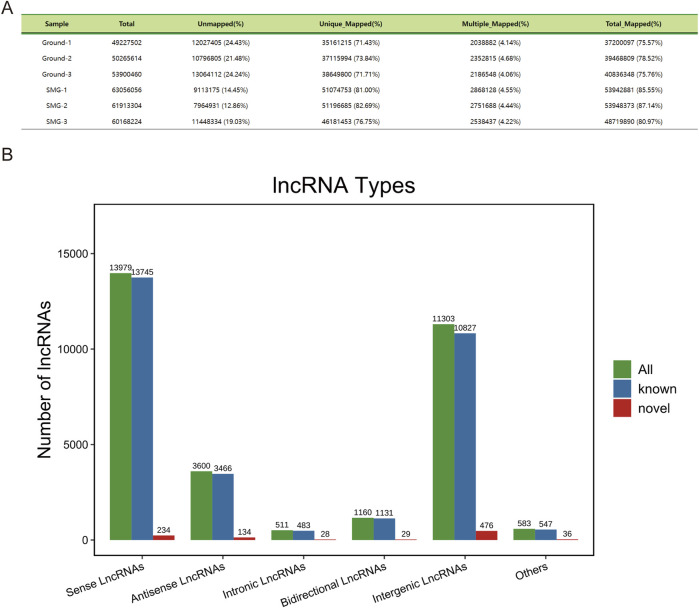
Sequencing data description and lncRNA characterization. **(A)** Comparison reference statistics for each sample. **(B)** Histogram of lncRNA types and components.

After ribosome removal, the reference genome comparison rate of each sample was greater than 75%, which indicated that ribosome removal was effective and that the data were high quality, providing a reliable database for subsequent bioinformatic analysis (Figure 1A). On the basis of the location of the newly identified lncRNAs on the genome relative to the protein-coding gene, we categorized the new lncRNAs into intergenic lncRNAs, bidirectional lncRNAs, intronic lncRNAs, antisense lncRNAs, and sense overlapping lncRNAs. In total, 476 intergenic lncRNAs, 29 bidirectional lncRNAs, 28 intronic lncRNAs, 134 antisense lncRNAs and 234 sense overlapping lncRNAs were identified (Figure 1B).

### Analysis of DElncRNA and mRNA expression levels in weightless bone loss

**FIGURE 2:**
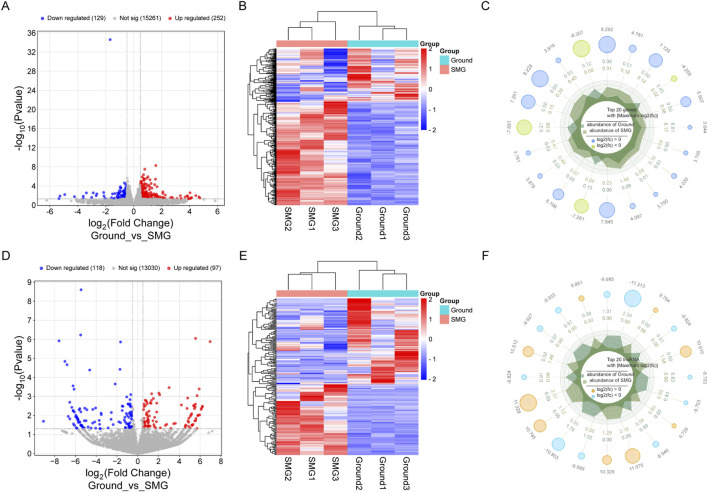
Analysis of differentially expressed genes in Ground and SMG groups. **(A)** DEmRNAs volcano map. **(B)** DEmRNAs heat map. **(C)** DEmRNAs radar plot. **(D)** DElncRNAs volcano map. **(E)** DElncRNAs heat map. **(F)** DElncRNAs radar map.

Through comparison of the DEGs between the ground and SMG groups, a total of 215 DElncRNAs and 381 DEmRNAs were identified, of which 97 lncRNAs were upregulated and 118 lncRNAs were downregulated. In addition, 252 upregulated mRNAs and 129 downregulated mRNAs were identified in the SMG group, and their specific differential expression distributions are shown in the volcano diagrams (Figures 2A, D). Moreover, the results of hierarchical clustering analysis revealed significant differences in the expression of DElncRNAs and DEmRNAs between the ground and SMG groups (Figures 2B, E). This study further exhibited the expression of the top 20 DElncRNAs and DEmRNAs, as shown in the radar plots (Figures 2C, F).

### DEmRNA function and signaling pathway enrichment analysis

**FIGURE 3:**
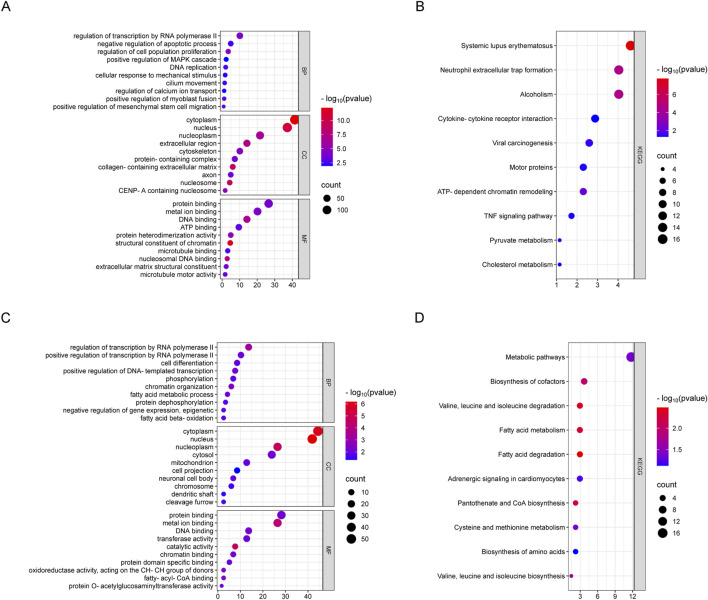
GO and KEGG enrichment analysis of differential genes in Ground group and SMG group. **(A)** Bubble diagram of DEmRNAs GO enrichment classification. **(B)** DEmRNAs KEGG enrichment bubble diagram. **(C)** DElncRNAs GO enrichment classification bubble diagram. **(D)** Bubble diagram of DElncRNAs GO enrichment classification.

LncRNAs primarily exert their functions by regulating the expression of coding genes. To further elucidate the functions of DElncRNAs and DEmRNAs in weightless bone loss, we performed GO function and KEGG pathway enrichment analyses for the DEmRNAs and DElncRNAs. GO enrichment analysis revealed that the and DEmRNAs were mainly enriched in the BP categories cell response to mechanical stimulation, the regulation of cell proliferation, the regulation of RNA polymerase II transcription and the regulation of calcium ion transport. In MFs, DEmRNAs are primarily involved in nucleosome DNA binding, microtubule motility and protein heterodimerization. In CCs, these molecules are mainly concentrated in the cytoplasm, nucleosome and extracellular matrix containing collagen. In addition, DElncRNAs are significantly enriched in cell differentiation, fatty acid metabolic process, epigenetic and phosphorylation during weightless bone loss. The bubble map shows 10 items that differ significantly from the GO results (Figures 3A, C). The results of the KEGG pathway enrichment analysis are shown in Figures 3B, D. DEmRNAs were significantly enriched in the regulation of the TNF signaling pathway, pyruvate metabolism, cholesterol metabolism, cytokine receptor interaction and other pathways. DElncRNAs are involved mainly in fatty acid metabolism, pantothenate and CoA biosynthesis, and biosynthesis of amino acids.

### LncRNA-mRNA coexpression network construction and correlation analysis

**FIGURE 4:**
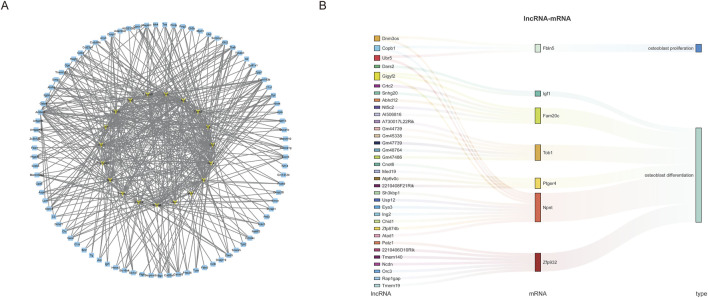
LncRNA-mRNA co-expression network construction. **(A)** The top 12 lncRNAs with the largest up- and downregulation folds were selected to construct the lncRNA-mRNA coexpression network diagram, with yellow representing lncRNAs and blue representing mRNAs. **(B)** Sankey diagram was used to demonstrate the coexpression and regulation of lncRNAs and mRNAs related to the function of osteoblast proliferation and differentiation.

To further explore the potential interactions between lncRNAs and mRNAs during weightless bone loss, we selected the top 12 lncRNAs with the largest upregulation and downregulation ratios among the DElncRNAs to construct the lncRNA-mRNA interaction network (Figure 4A). Coexpression network analysis revealed a total of 106 nodes: 19 lncRNAs and 87 mRNAs. The network diagram illustrates that multiple lncRNA nodes are connected to multiple mRNA nodes, forming a complex regulatory network. In addition, to identify lncRNAs related to osteoblast proliferation and differentiation, we further analyzed the mRNAs and lncRNAs via trans-association analysis. As Figure 4B shows, a total of 34 DElncRNAs and 7 DEmRNAs were associated with osteoblast proliferation and differentiation. These results suggest that these genes may play important regulatory roles in the occurrence and development of weightless bone loss.

### qRT-PCR validation of DElncRNAs and DEmRNAs in weightless bone loss

**FIGURE 5:**
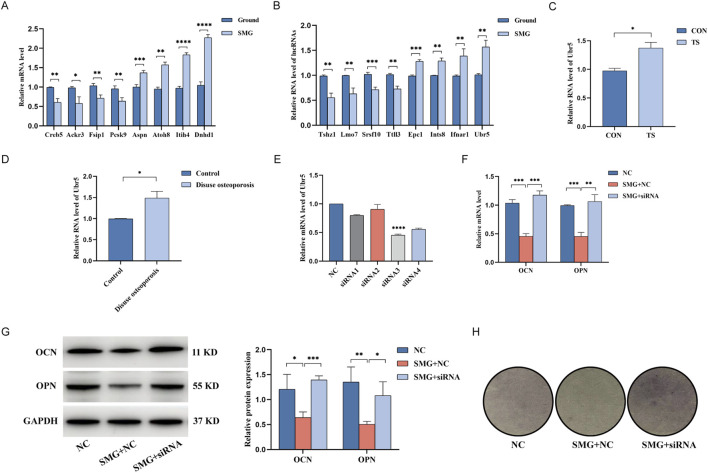
qRT-PCR to verify the expression of differential genes and the effect of lncRNA Ubr5 on the osteogenic and differentiation function of BMSCs in weightless bone loss. **(A)** Expression levels of lncRNA in Ground and SMG groups. **(B)** Expression levels of mRNA in Ground and SMG groups. **(C)** Expression levels of lncRNA Ubr5 in the bone tissues of mice in CON and TS groups. **(D)** Expression levels of lncRNA Ubr5 in the bone tissues of clinical patients in Control and Disuse osteoporosis groups. **(E)** qRT-PCR analysis for expressions of lncRNA Ubr5 after siRNA infection. **(F)** qRT-PCR analysis for expressions of OCN and OPN at the RNA level after knockdown of lncRNA Ubr5 and simulated weightlessness for 48 h. **(G)** Western blot analysis of the expression of OCN and OPN protein level after knockdown of lncRNA Ubr5 and simulated weightlessness for 48 h. **(H)** ALP staining assay to analyze the osteogenic differentiation effect. *P < 0.05; **P < 0.01; ***P < 0.001; ****P < 0.0001.

To validate the differential gene expression levels found via Illumina sequencing, we randomly selected 8 lncRNAs and 8 mRNAs and measured their expression in both groups via qRT-PCR. The results revealed that all the selected lncRNAs and mRNAs showed significant differences (Figures 5A, B). The qRT-PCR results were consistent with the sequencing results confirmed the reliability and accuracy of the sequencing data. Some key genes exist in the coexpression network, which further confirms the reliability of the coexpression network analysis. Notably, the qRT-PCR results revealed the most significant upregulation of the lncRNA Ubr5, and Figure 5B shows that the lncRNA Ubr5 may play an important role in both the proliferation and differentiation of osteoblasts. Furthermore, we examined the expression levels of the lncRNA Ubr5 in the mouse tail suspension model (Figure 5C) and the human disuse osteoporosis model (Figure 5D) via qRT-PCR. The results revealed that lncRNA Ubr5 expression was significantly increased in both the tail suspension group and the disuse osteoporosis group, which was consistent with the findings in our cell model simulating the effect of weightlessness. Therefore, we identified the lncRNA Ubr5 as a core gene that deserves further in-depth investigation due to its potential significance in weightless bone loss.

### Knockdown of Ubr5 can induce bone formation and osteogenic differentiation of BMSCs

To investigate the biological function of Ubr5 in weightless bone loss, we measured the expression levels of Ubr5 in the cells, mouse bone tissue and lumbar vertebrae of patients with disuse osteoporosis via qRT-PCR. Compared with that in the ground group, the expression level of Ubr5 was significantly higher in the SMG group, tail suspension group and disuse osteoporosis group. Therefore, we synthesized four siRNAs for Ubr5 and transfected them into BMSCs. Subsequently, qRT-PCR was used to verify the knockdown efficiency, and siRNA3 was identified as the best sequence (Figure 5E). To further study the effects of Ubr5 on the bone formation and osteogenic differentiation of BMSCs, we assessed the protein expression levels of OCN and OPN via Western blotting (Figure 5G). The results revealed that the protein expression of OCN and OPN was lower in the SMG group than in the ground group and that the expression levels of these proteins were partially restored after Ubr5 was knocked down. The results of the qRT-PCR were also consistent with these findings (Figure 5F). In addition, we examined the effect of Ubr5 knockdown on the osteogenic differentiation of BMSCs via an ALP staining kit. Compared with that in the ground group, the degree of staining was significantly lower in the SMG group, while the degree of staining in the SMG + siRNA group recovered to the same level as that in the ground group (Figure 5H). These results suggest that Ubr5 knockdown can promote the bone formation and osteogenic differentiation of BMSCs, highlighting Ubr5 as a potential therapeutic target for weightless bone loss.

### Knockdown of Ubr5 can promote the proliferation and inhibit the apoptosis of BMSCs

**FIGURE 6:**
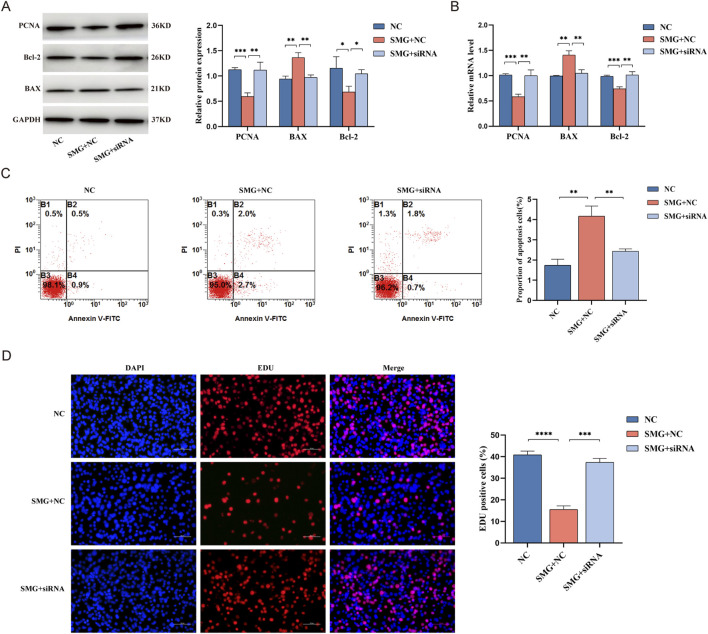
Effects of lncRNA Ubr5 on the proliferation and apoptotic of BMSCs in weightless bone loss. **(A)** Western blot analysis of the expression of PCNA, BAX and Bcl-2 at the protein level after knockdown of lncRNA Ubr5 and simulated weightlessness for 48 h. **(B)** qRT-PCR analysis of PCNA, BAX and Bcl-2 expression at the RNA level after knockdown of lncRNA Ubr5 and simulated weightlessness for 48 h. **(C)** Evaluating apoptosis by flow cytometry. **(D)** Cell proliferation was assessed using EDU assays; Scale bars: 100 μm, *P < 0.05; **P < 0.01; ***P < 0.001; ****P < 0.0001.

To investigate the effect of Ubr5 on the proliferation and apoptosis of BMSCs, we measured the protein expression levels of PCNA、BAX and Bcl-2 via Western blotting to explore the effect of Ubr5 on the proliferation and apoptosis. Compared with that in the ground group, the protein expression of BAX was increased, and the protein expression of PCNA and Bcl-2 was decreased in the SMG group. However, the expression levels of these proteins were partially reversed upon Ubr5 knockdown (Figure 6A). The results of qRT-PCR were consistent with these findings (Figure 6B). In addition, the EDU results showed that weightlessness inhibited the proliferation of BMSCs, while Ubr5 knockdown effectively rescued this inhibition (Figure 6D). We further examined the effect of Ubr5 on the percentage of apoptotic cells via flow cytometry (Figure 6C). The results revealed that the total apoptosis rate of the BMSCs in the SMG group was increased by approximately 3.36-fold compared with that in the ground group, whereas the total apoptosis rate in the SMG + siRNA group decreased significantly, indicating that simulated weightlessness for 48 h significantly promotes the apoptosis of BMSCs, and this effect can be partially reversed by Ubr5 knockdown. These results collectively demonstrate that Ubr5 knockdown can promote the proliferation and inhibit the apoptosis of BMSCs, suggesting that Ubr5 plays a critical role in mediating the adverse effects of weightlessness on these cells.

## Discussion

In the weightless environment of space, astronauts undergo substantial bone loss due to the absence of gravity, which poses a severe threat to their health and the success of their space missions. In recent years, scholars from different countries have proposed various potential mechanisms of weightless bone loss, provideing an important theoretical basis for further research in this field. Some studies (Vico et al., 2000; Dallas et al., 2013) have suggested that weightless bone loss primarily occurs because the mechanical load on bones is significantly reduced under weightlessness. Additionally, some scholars have proposed (Hargens and Richardson, 2009) that the redistribution of body fluids in the absence of gravity may be one of the factors leading to bone loss. Further studies (Arfat et al., 2014) have also indicated that weightless bone loss may result from a dynamic imbalance between osteoclast-mediated bone resorption and osteoblast-mediated bone formation. Notably, studies by Chinese scientists have revealed (Sun et al., 2016) that osteoclasts in a weightless environment can transfer small nucleic acid molecules affected by gravity to osteoblasts through exosomes, inhibiting the function of osteoblasts and resulting in the occurrence of osteoporosis. In weightlessness, the activity of osteoblasts may decrease, leading to less new bone formation. Osteoblasts are responsible for the synthesis and secretion of bone matrix, but their function may be inhibited under weightless conditions, possibly due to changes in signaling pathways caused by reduced mechanical stimulation; Bone cells may influence bone formation and bone resorption by altering the signaling molecules they secrete, such as sclerostin and RANKL. Under weightless conditions, osteocytes may increase the expression of sclerostin, thereby inhibiting the activity of osteoblasts and reducing bone formation. In addition, the activity of osteoclasts may be increased in a weightless environment, which may be related to the increase of RANKL secreted by bone cells, a signaling molecule that promotes osteoclast formation, leading to accelerated bone resorption. In addition, BMSCs (Lü et al., 2019; Chen et al., 2015) are a class of adult stem cells characterized by their self-renewal capacity and multidirectional differentiation potential that can differentiate into osteoblasts and other cell types. Studies have demonstrated (Dai et al., 2007; Zayzafoon et al., 2004) that the differentiation tendency of BMSCs is significantly altered under weightlessness, inhibiting their differentiation into osteoblasts while promoting their differentiation into adipocytes, leading to bone loss. However, the gene expression profile and the underlying molecular mechanism of weightless bone loss remain unclear. This study investigated the molecular mechanism of the weightless bone loss gene expression profile and explored potential therapeutic targets through bioinformatic methods. Our findings not only provide a novel perspective for understanding the pathological mechanisms of weightless bone loss but also lay the foundation for identifying potential therapeutic targets. This work offers a foundation for the development of effective intervention strategies to address this critical issue in the future.

Noncoding RNAs (Rinn and Chang, 2012; Mercer et al., 2009), which include long noncoding RNAs (lncRNAs) and small RNAs, represent a vast class of RNA molecules that do not encode proteins. These molecules play crucial roles in biological processes, such as gene expression regulation, cell development, and disease occurrence. At present, lncRNAs has become a focal point in the biomedical field, but the biological functions and molecular mechanisms of most lncRNAs remainl unclear. Recent studies (Liu et al., 2024a; Li, 2023) have shown that lncRNAs can play regulatory roles in multiple stages of bone formation, including cell proliferation, differentiation and apoptosis. These lncRNAs have been closely related to osteoporosis and other bone-related diseases. However, studies on the expression profile and function of lncRNAs in weightless bone loss are still limited. Comprehensive analyses of lncRNAs and mRNAs in this context are scarce, which to some extent constrains the development of prevention and treatment strategies for weightless bone loss. In this study, the expression profiles of lncRNAs and mRNAs associated with weightless bone loss were constructed via high-throughput sequencing and bioinformatic methods with BMSCs from the ground group and the SMG group, and a coexpression network of lncRNAs and mRNAs was constructed to explore their functions and interactions. These findings provide a foundation for further identifying lncRNAs that play important roles in the occurrence and progression of weightless bone loss and provide potential targets for future prevention and control measures.

In this study, a 2D-gyroscope was used to simulate the effects of BMSCs in a weightless environment, and the expression profiles of lncRNAs and mRNAs associated with weightless bone loss were analyzed via high-throughput sequencing. A total of 215 lncRNAs and 381 mRNAs were identified. Compared with the ground control group, the SMG group exhibited 252 upregulated and 129 downregulated mRNAs, as well as 97 upregulated and 118 downregulated lncRNAs. Given that the function of lncRNA is closely related to their downstream mRNAs, we performed GO function and KEGG pathway enrichment analyses on the DEmRNAs to further elucidate the roles of these DElncRNAs and DEmRNAs in the SMG group. GO enrichment analysis revealed that the weightless environment had a significant effect on the biological process of the cell mechanical response, especially the osteoblast mechanical response process. Previous studies (Dallas et al., 2013) have demonstrated that the loss of mechanical stimulation of osteoblasts in a weightless environment leads to the inhibition of their proliferation and differentiation, thus hindering the formation and repair of bone tissue and leading to bone loss. These findings are consistent with the results of the present study, highlighting the critical role of mechanical stimuli in maintaining bone health. In addition, GO analysis revealed that microtubule movement plays a key role in molecular function. Previous studies (Bonewald and Johnson, 2008) have shown that microtubules are crucial for cellular responses to mechanical signals. Osteoblasts rely on microtubules to sense and transmit mechanical stress, which is essential for bone growth and maintenance. However, microtubule motor activity is weakened in a weightless environment, which leads to impaired proliferation, differentiation and mineralization of osteoblasts. This ultimately results in reduced bone density and the development of osteoporosis. KEGG pathway enrichment analysis revealed that the DEmRNAs participated in multiple biological pathways. Previous studies (Boyce and Xing, 2008; Sims and Walsh, 2012) have reported that the TNF signaling pathway plays a role in postmenopausal osteoporosis and systemic lupus erythematosus (SLE)-associated osteoporosis. Our study further revealed that this pathway similarly mediates the occurrence of osteoporosis under weightless conditions, suggesting that these enriched pathways may play important roles in the occurrence and development of weightless bone loss. To explore the regulatory functions and mechanisms of lncRNAs in detail, we selected the 12 lncRNAs with the highest levels of upregulation and downregulation and constructed a lncRNA-mRNA coexpression network. Coexpression network analysis revealed that 87 differentially expressed mRNAs were coexpressed with 19 DElncRNAs. This network provides a noval perspective for studying the function of new lncRNAs in weightless bone loss and may provide a potential molecular marker for the prevention and treatment of weightless bone loss. To further investigate lncRNAs associated with osteoblast proliferation and differentiation, we constructed a specialized lncRNA-mRNA coexpression network related to osteoblast proliferation and differentiation. 7 candidate mRNAs and 34 candidate lncRNAs, including Npnt, which plays a key role in bone tissue development, homeostasis, and repair, were identified. Previous studies (Sun et al., 2018; Klein-Nulend et al., 2005) have demonstrated that Npnt regulates signal transduction by interacting with integrin α8β1, thus affecting the proliferation and differentiation of osteoblasts. Decreased Npnt expression was associated with decreased bone mass and osteogenic marker expression, suggesting that Npnt plays an important role in bone tissue homeostasis and repair. In addition, changes in Npnt expression have been shown to be associated with bone diseases such as osteoporosis and cancer bone metastases. The regulatory functions of Npnt provide new potential therapeutic targets for the treatment of these bone-related conditions, further underscoring its significance in bone health and disease.

Through Pearson correlation analysis, we subsequently identified that lncRNAs are coexpressed with Npnt and highlighted that Ubr5 may play an important role in both the proliferation and differentiation of osteoblasts, making it a highly valuable target for further research. Ubr5 (Hu and Chen, 2024) is a key E3 ubiquitin ligase that plays a crucial role in cell biology by regulating protein degradation and signaling pathways. Initially, Ubr5 was identified as a tumor suppressor in breast cancer cells (Xiang et al., 2022). Studies have shown that Ubr5 expression is elevated in various tumor types and is closely related to tumorigenesis. Ubr5 is considered a risk factor in osteosarcoma and has a major effect on the immune microenvironment. However, no studies on Ubr5 in bone-related diseases have been reported. Therefore, this study utilized cell biology and bioinformatic methods to explore the function of Ubr5 in weightless bone loss and its potential mechanism of action. To investigate the biological function of Ubr5 in weightless bone loss, we conducted several functional experiments in BMSCs. The results revealed that weightlessness significantly reduced the proliferation and osteogenic differentiation of BMSCs while promoting their apoptosis. Importantly, these adverse effects were reversed by silencing Ubr5. These results suggest that Ubr5 plays a critical role in weightless bone loss and may be a potential therapeutic and preventive target for this condition.

In this study, 8 lncRNAs with large variations were randomly selected from 215 DElncRNAs and verified their expression levels by qRT-PCR. The results were consistent with the sequencing results, confirming the reliability of our findings. Teashirt zinc finger homeobox 1 (TSHZ1) (Chaimowicz et al., 2019) is a transcription factor that contains a zinc finger and a homologous heterodimeric structural domain. It is involved in various biological processes. Studies have shown that TSHZ1 is abnormally expressed in a variety of cancers, especially colon cancer, where it may play a role in colon cancer development as the serum marker cancer antigen 33. Research by Nathalie Core (Coré et al., 2007) revealed that TSHZ1 is essential for the development of the axial bones, the soft palate, and the middle ear in mice and is involved in the regulatory pathways of bone morphogenesis. Therefore, TSHZ1 plays an important role in bone development. LIM domain only 7 (LMO7) is an critical regulator of skeletal muscle development. Liu (Gomes et al., 2021) et al. reported that LMO7 plays a key role in muscle development in mice, and LMO7 knockout mice exhibit muscle atrophy similar to Emery-Dreifuss muscular dystrophy. In addition, LMO7 is closely related to the progression and metastasis of pancreatic cancer. Loss of LMO7 function can induce cycle arrest and apoptosis of pancreatic cancer cells, making LMO7 has become a promising target for pancreatic cancer therapy (Liu et al., 2021). Serine/arginine-rich splicing factor 10 (SRSF10) (Cai et al., 2024) is a splicing factor that plays a key role in RNA splicing. Li J. et al. (2023) reported that SRSF10 affects the proliferation of neural precursor cells and cortical neurogenesis by regulating the PI3K-AKT-mTOR-CCND2 signaling pathway and the selective splicing of the Nasp gene. TTLL3 (Pathak et al., 2011) is a microtubulin tyrosine ligase involved in the post-translational modification of microtubulin. Wloga et al. (2009) demonstrated that TTLL3 is essential for the formation and maintenance of cilia. Given that bone cells such as osteoblasts and osteoclasts have cilia, TTLL3 may be indirectly influence bone health by modulating the signaling pathways in these cells. IFNAR1(Li et al., 2023b) is a key element of type I interferon signaling in the immune system, activates the JAK-STAT signaling pathway, and plays an vital role in cellular immunity and the antiviral response. Integrator complex subunit 8 (INTS8) (Zhou et al., 2021) is a component of the RNA polymerase II complex, which is involved in the cleavage and transcription of small nuclear RNA. Recent studies have revealed that the expression of INTS8 is upregulated in a variety of cancers, especially intrahepatic cholangiocarcinoma, and is closely related to poor prognosis. Enhancer of polycomb 1 (EPC1) is a protein associated with the polycomb complex that regulates gene silencing and stem cell differentiation. Recent studies (Liu et al., 2024b) have shown that EPC1 plays a role in the proliferation of hematopoietic stem cells and progenitor cells by regulating the acetylation of histone H3 and the activity of the S-succinyltransferase dihydrolipoic acid. In addition, EPC1 is associated with acute myeloid leukemia (AML), where it may maintain its carcinogenic potential by inhibiting MYC accumulation and the apoptosis of AML cells. However, the function and role of these lncRNAs in weightless bone loss have not been reported. Therefore, our research group plans to explore the potential mechanism of these lncRNAs in weightless bone loss in depth in the future.

In summary, this study has identified numerous DElncRNAs and DEmRNAs as potential candidate molecules for the study of weightless bone loss. For the first time, bioinformatic methods were used to construct a transcriptomic lncRNA and mRNA coexpression network of weightless bone loss, which contributed to a deeper understanding of the potential functions of these DEGs. Through functional clustering and enrichment analysis of DEmRNAs, we identified functional modules associated with osteoblast proliferation and differentiation. In subsequent analyses, Ubr5 was selected as a key lncRNA, and its important role in weightless bone loss was proven via *in vitro* experiments, providing novel clues for exploring the mechanism of weightless bone loss. However, this study also has certain shortcomings and limitations. First, 2D-gyroscope simulated weightlessness model, mouse tail suspension simulated microgravity model, and clinical patient disuse osteoporosis model may not be able to fully replicate the complex and multifaceted conditions experienced by the human body in a real spatial environment, a limitation that may affect the broader applicability of our findings. Second, the number of patients meeting the inclusion criteria in this study was limited, with only six patients with disuse osteoporosis being recruited; thus, the study results still need to be further verified in a larger sample size. Finally, the findings presented in this study are a step towards understanding the complex regulatory network of weightlessness induced bone loss, but further detailed studies are needed to fully elucidate the mechanisms, in particular the role of lncRNAs.In future studies, we intend to begin investigating the specific mechanisms of lncRNA in bone metabolism, which can provide key insights into the underlying biology and potential therapeutic targets.

## Conclusion

In this study, we found that lncRNA and mRNA expression was significantly greater in the SMG group than in the ground group. We identified and validated 8 key lncRNAs and constructed a lncRNA-mRNA coexpression network, which lays the foundation for further elucidation of the molecular mechanisms underlying the development of weightless bone loss. In addition, we constructed a coexpression network related to osteoblast proliferation and differentiation and identified the lncRNA Ubr5 as a key lncRNA, which provides a new reference for studying the occurrence and development of weightless bone loss at the cellular level. These findings not only broaden our understanding of the role of lncRNAs in the regulation of bone metabolism but also provide potential molecular markers and intervention targets for the study of molecular mechanisms and prevention and treatment of weightless bone loss.

## Data Availability

The data presented in the study are deposited in the NCBI repository, accession number SUB11153018.
