# Supplementary material for: lncRNA Ubr5 promotes BMSCs apoptosis and inhibits their proliferation and osteogenic differentiation in weightless bone loss
Source: Front Cell Dev Biol. 2025 Apr 2;13:1543929. doi: 10.3389/fcell.2025.1543929 (PMC11999945; doi:10.3389/fcell.2025.1543929)
Supplement: Supplementary file 1 [file Table1.docx]

**Supplementary Table 1. Basic patient information**

|  | Name | Sex | Age | BMD |
| --- | --- | --- | --- | --- |
| Disuse Osteoporosis | 1 | Male | 65 | -3.6 |
|  | 2 | Male | 67 | -2.5 |
|  | 3 | Male | 58 | -2.2 |
|  | 4 | Male | 54 | -2.8 |
|  | 5 | Male | 60 | -1.9 |
|  | 6 | Male | 52 | -2.1 |
| Normal bone mineral density | 1 | Male | 50 | -0.5 |
|  | 2 | Male | 48 | 0.2 |
|  | 3 | Male | 52 | 1.1 |
|  | 4 | Male | 55 | -0.2 |
|  | 5 | Male | 46 | -0.3 |
|  | 6 | Male | 53 | 1.5 |
