# Supplementary figures and images for: lncRNA Ubr5 promotes BMSCs apoptosis and inhibits their proliferation and osteogenic differentiation in weightless bone loss
Source: Front Cell Dev Biol. 2025 Apr 2;13:1543929. doi: 10.3389/fcell.2025.1543929 (PMC11999945; doi:10.3389/fcell.2025.1543929)

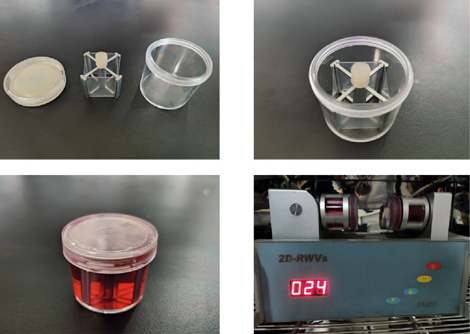

Supplement: Supplementary file 2 [file Image3.tif]

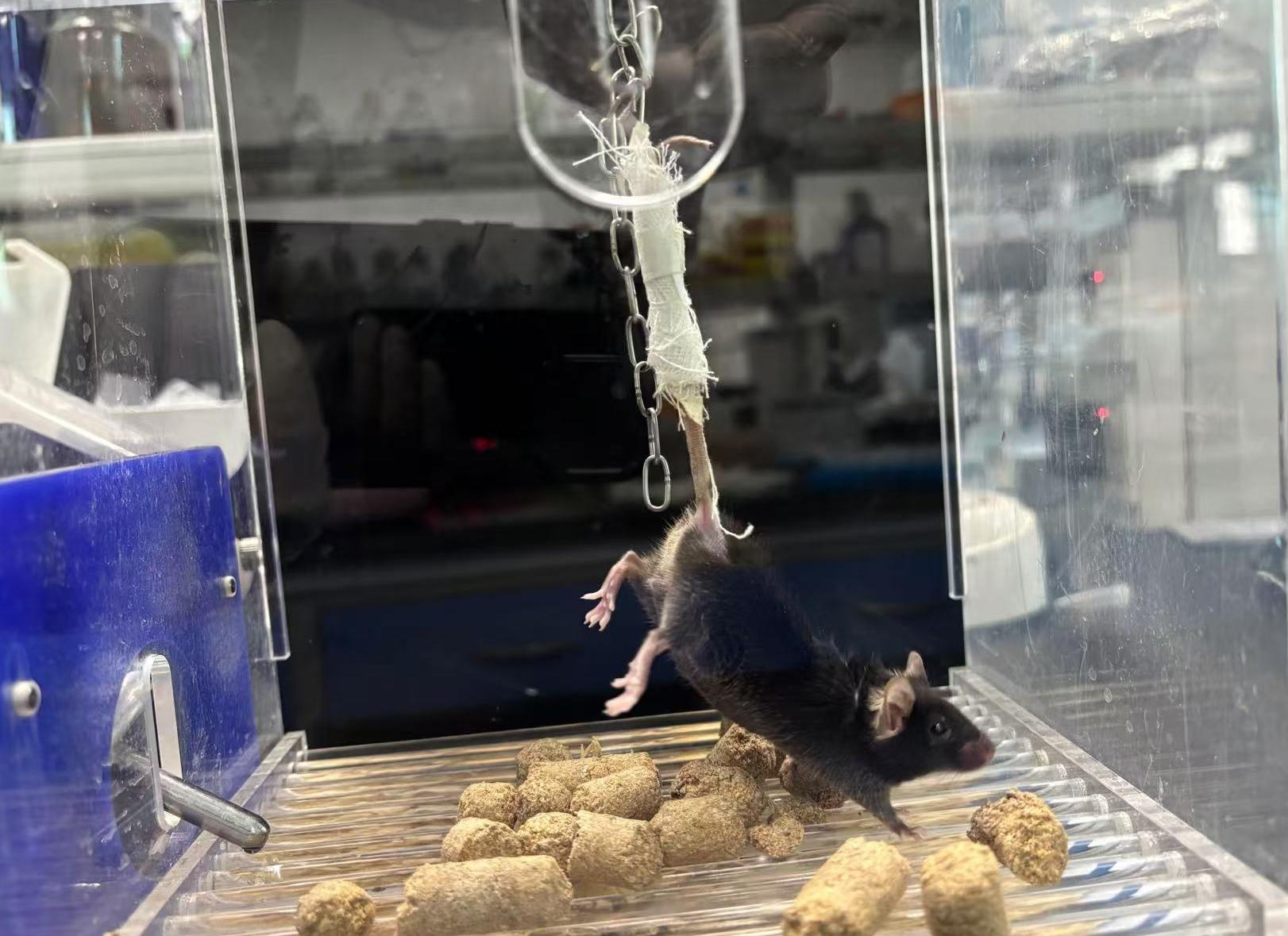

Supplement: Supplementary file 3 [file Image2.jpeg]

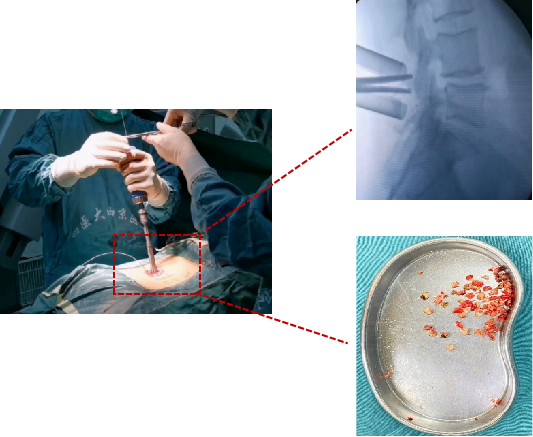

Supplement: Supplementary file 4 [file Image1.tif]
